# Cardiological Challenges Related to Long-Term Mechanical Circulatory Support for Advanced Heart Failure in Patients with Chronic Non-Ischemic Cardiomyopathy

**DOI:** 10.3390/jcm12206451

**Published:** 2023-10-10

**Authors:** Michael Dandel

**Affiliations:** German Centre for Heart and Circulatory Research (DZHK), 10785 Berlin, Germany; mdandel@aol.com

**Keywords:** ventricular assist devices, non-ischemic cardiomyopathy, chronic heart failure, myocardial recovery, weaning from long-term mechanical support

## Abstract

Long-term mechanical circulatory support by a left ventricular assist device (LVAD), with or without an additional temporary or long-term right ventricular (RV) support, is a life-saving therapy for advanced heart failure (HF) refractory to pharmacological treatment, as well as for both device and surgical optimization therapies. In patients with chronic non-ischemic cardiomyopathy (NICM), timely prediction of HF’s transition into its end stage, necessitating life-saving heart transplantation or long-term VAD support (as a bridge-to-transplantation or destination therapy), remains particularly challenging, given the wide range of possible etiologies, pathophysiological features, and clinical presentations of NICM. Decision-making between the necessity of an LVAD or a biventricular assist device (BVAD) is crucial because both unnecessary use of a BVAD and irreversible right ventricular (RV) failure after LVAD implantation can seriously impair patient outcomes. The pre-operative or, at the latest, intraoperative prediction of RV function after LVAD implantation is reliably possible, but necessitates integrative evaluations of many different echocardiographic, hemodynamic, clinical, and laboratory parameters. VADs create favorable conditions for the reversal of structural and functional cardiac alterations not only in acute forms of HF, but also in chronic HF. Although full cardiac recovery is rather unusual in VAD recipients with pre-implant chronic HF, the search for myocardial reverse remodelling and functional improvement is worthwhile because, for sufficiently recovered patients, weaning from VADs has proved to be feasible and capable of providing survival benefits and better quality of life even if recovery remains incomplete. This review article aimed to provide an updated theoretical and practical background for those engaged in this highly demanding and still current topic due to the continuous technical progress in the optimization of long-term VADs, as well as due to the new challenges which have emerged in conjunction with the proof of a possible myocardial recovery during long-term ventricular support up to levels which allow successful device explantation.

## 1. Introduction

For patients with advanced (stage D) chronic heart failure (HF) refractory to guideline-directed pharmacological treatment as well as to both device and surgical optimization therapies (e.g., cardiac resynchronization therapy, implantable cardioverter–defibrillator devices, transcatheter or surgical treatment of severe secondary mitral regurgitation), long-term mechanical circulatory support (MCS) by a left ventricular assist device (LVAD), with or without an additional temporary or long-term right ventricular (RV) support, is a well-established life-saving therapeutic option [1]. Implantation of a durable VAD as a bridge-to-transplant (BTT) or, for patients not deemed to be heart transplant (HTx) candidates, as a destination therapy (DT) should be considered in carefully selected patients with NHYA class IV symptoms despite maximal ambulatory medical therapy, as well as in those who are dependent on intravenous administration of inotropes or temporary MCS [1]. 

The survival benefit for durable LVAD support has progressively improved. Recent trials with newer generation LVADs revealed two-year survival rates above 80%, which were similar to those reported for the early survival after HTx [1,2]. The latest findings have also shown improved mean survival (i.e., >4 years) for patients with DT-LVADs [1,3]. In many studies, durable LVAD support was also associated with highly relevant functional and quality of life improvement, even though the patients remain tethered to external electrical power supplies via a percutaneous lead [1,2,4,5]. Elevated central venous pressure, pulmonary hypertension, and coagulopathy were found to be particularly linked to poorer outcomes [1,6,7,8]. 

**Figure 1:**
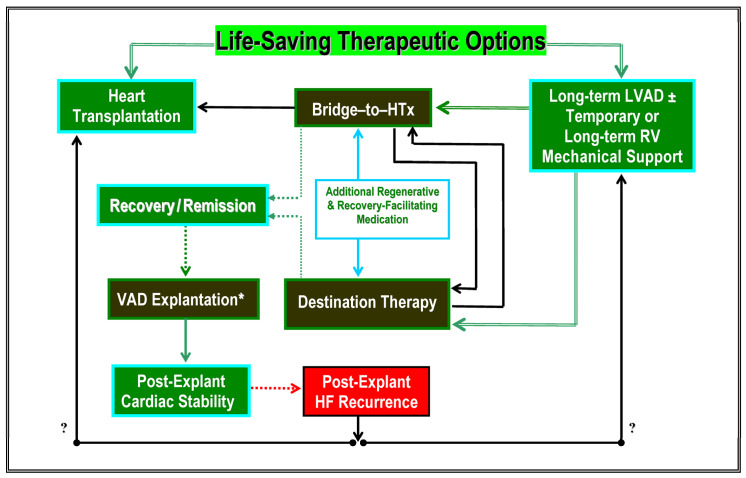
Life-saving therapeutic options for patients with refractory end-stage heart failure. HTx, heart transplantation; LVAD, left ventricular assist device; RV, right ventricle; VAD, ventricular assist device; HF, heart failure. Double line arrows indicate initial therapeutic goals; dotted arrows indicate lower probability. * VAD explantation can be successful even after incomplete cardiac recovery. ? indicates that in patients with post-explant HF recurrence, two options remain available (i.e., either HTx or long-term support by a new VAD).

It is important to note that the BTT and DT designations are not unchangeable. Patients may cross over from one group to the other as their clinical state improves or deteriorates, or if a formerly transplant-eligible patient must remain on indefinite VAD support because of newly arisen risks related to different co-morbidites (Figure 1).

This review article aimed to provide an updated theoretical and practical basis for those engaged in this highly demanding and still current topic due to the continuous technical progress in the optimization of MCS devices, as well as due to the new challenges which have emerged in conjunction with the increasing evidence of a possible myocardial recovery during long-term VAD support up to levels which allow successful device explantation. 

## 2. Non-Ischemic Cardiomyopathy-Induced Heart Failure

Cardiomyopathies are a heterogeneous group of myocardial diseases that cause structural and functional damages which induce mechanical and/or electrical cardiac dysfunction, which, in turn, result in dilated, hypertrophic, or restrictive pathophysiology [9,10]. The generic term “non-ischemic cardiomyopathy” (NICM) includes all causes of myocardial structural and functional alterations of various etiologies that are not primarily caused by coronary artery disease or abnormal loading conditions [11]. 

### 2.1. Etiopathogenic and Pathophysiological Particularities of NICM 

Chronic inflammatory cardiomyopathy, a progressive disorder most often either directly induced or associated with different virus infections, is the most common cause of advanced HF in patients with NICM [11,12,13]. In these patients (particularly in the presence of genetic susceptibility to inflammation and/or myocardial predisposition to maladaptive remodeling), chronic inflammation-induced myocardial alterations trigger LV remodeling, leading more often to dilated cardiomyopathy (DCM) or, alternatively, to a predominant hypokinetic (LVEF <45%) non-dilated phenotype of cardiomyopathy [12]. 

**Figure 2:**
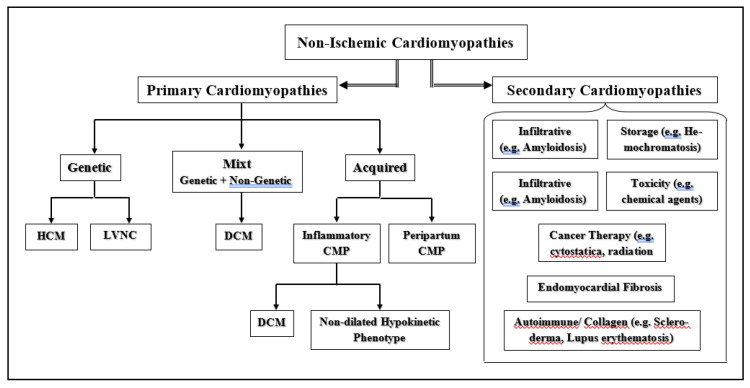
Classification of the heterogenous group of non-ischemic cardiomyopathies. HCM, hypertrophic cardiomyopathy; LVNC, left ventricular noncompaction; DCM, dilated cardiomyopathy; CMP, cardiomyopathy.

Given that non-ischemic DCM results from a variety of interconnected genetic and acquired triggers, patients with DCM are by no means a homogenous group (Figure 2) [14,15]. A recent study identified four different DCM phenogroups associated with significant differences in clinical presentation, underlying molecular profiles, and outcome, which could facilitate more personalized treatment approaches [16]. The main pathophysiological mechanisms which are involved in the development and progression of DCM are related to the deleterious impact of the primary myocardial damage (produced most often by a viral infection) and to subsequent ongoing autoimmune insults triggered by myocarditis-inducing agents [17,18]. The fact that many autoimmune reactions in DCM appear to be virus-triggered indicates that the two pathophysiological mechanisms are usually interconnected [18]. In about 30% of all DCM cases, a genetic predisposition to autoimmune reactions appeared to be related to the development of the myocardial alterations [19]. Several alterations of cell-mediated immunity detected in myocardial biopsies from DCM patients suggest the pathogenetic involvement of cellular autoimmunity in the disease [20]. A crucial argument for a relevant pathogenetic role of autoimmunity in the development and progression of DCM was, however, the frequent detection of autoantibodies (AABs) against structural and contractile myocardial proteins, as well as proteins of energy metabolism/transfer, ion channels, and sarcolemmal receptors in patient sera [20,21]. Several cardiac-specific AABs (particularly β_1_-AABs, α-myosin-AABs, and cardiac troponin I AABs) revealed a particularly relevant pathologic potential [22,23]. It has been suggested that myocardial tissue damage may induce the release of intracellular proteins that can become “self-antigens” able to trigger humoral responses leading to the production of AABs [24].

Among the AABs which were more often detected in serum from NICM patients, those against the β_1_-adreno-receptors (β_1_-AABs) proved to have a particularly high pathophysiological relevance in both the development and the progression of the disease [23,25,26]. Whereas β_1_-AABs are rarely detectable in healthy persons and only in 10–13% of patients with ischemic cardiomyopathy, 26–60% of IDCM patients manageable with guideline-directed pharmacological therapy tested positive for β_1_-AABs [19,27,28,29,30]. In patients with end-stage NICM who required the implantation of a VAD for durable support, the prevalence of those testing positive for functional β_1_-AABs exceeded 80% [31,32]. This suggests that the aggravation of myocardial dysfunction in NICM could be closely connected to the presence of β_1_-AABs. Indeed, the presence β_1_-AABs appeared to be associated with a three-fold increase in all-cause and cardiovascular mortality risk in DCM [30]. The pathogenic potential of β_1_-AABs was also underpinned by studies in which peptides corresponding to the second extracellular loop of human β_1_-AABs were able to induce myocardial changes in animals which were similar to those found in DCM patients [22]. Based on clinical and experimental observations, two decades ago it was already postulated that in β_1_-AAB-positive DCM patients, cardiomyopathy can be considered as a β_1_-adrenoreceptor-targeted autoimmune disease [22] The detrimental impact of β_1_-AABs on cardiac function in patients with NICM was also revealed indirectly by several clinical studies which showed that in HTx candidates with non-ischemic DCM, the removal of β_1_-AABs by immunoadsorption (IA) can not only ameliorate the clinical symptoms, but also significantly improve both the LVEF and long-term HTx/VAD-free survival [18,33,34]. Another important observation which supports the pathogenic importance of β_1_-AABs in non-ischemic DCM was the disappearance of these AABs in 97% of the initially β_1_-AAB-positive patients with relevant myocardial recovery during prolonged cardiac mechanical support, which has proved to be sufficient to enable the explantation of the VAD [32].

In contrast to a physiologically regulated signal cascade, the binding of β_1_-AABs to β_1_- adrenoreceptors (β_1_-ARs) leads to persistent overstimulation of these receptors, which is associated with lack of down-regulation and desensitization of the β-adrenergic reaction cascade [35]. The chronic over-stimulation of the β_1_-ARs causes cardiomyocyte damage related mainly to the induced intra-cellular Ca^2+^-overload and the reduction in β_1_-AR expression [35,36]. In addition, the β_1_-AABs can also induce a serum level-dependent increase in the myocyte apoptosis rate [37,38]. Given the strong evidence regarding the relevant pathogenetic involvement of β_1_-AABs in the development and progression of non-ischemic DCM, the beneficial results obtained in studies evaluating the efficacy of β_1_-AAB removal by IA are not surprising [28,33]. The fact that, in HTx candidates with high β_1_-AAB serum levels, selective β_1_-AAB removal by specific IA and unselective IgG removal by non-specific IA had similar beneficial effects suggests that although β_1_-AABs are not the only AABs that could be involved in the pathogenesis of non-ischemic DCM, their pathogenic impact is of particular relevance [33]. This is also supported by the observation that the redetection of β_1_-AABs in some of the patients with relevant cardiac improvement after β_1_-AAB removal coincided with renewed deterioration of cardiac function [33]. 

Removal of AABs involved in the development and progression of NICM of different origins can spare some of the potential HTx candidates from long-term VAD support during the waiting time on the transplantation list or, for some of those not eligible for HTx, they may delay the need for a VAD as DT. In a study which included 108 β_1_-AAB-positive HTx candidates with end-stage non-ischemic DCM (LVEF <30%) who underwent unspecific IA between 1995 and 2005, removal of the β_1_-AABs was followed by a significant increase in the LVEF and a 3.7 times higher probability for five-year HTx/VAD-free survival compared to another β_1_-AAB-positive patient group with the same diagnosis and similar morphological and functional cardiac alterations at the baseline but without any subsequent removal of the β_1_-AABs [33]. Given that the IA can safely be performed without any complications and the high prevalence of responders to this therapy (i.e., 79.6%), as well as the high five-year post-IA HTx/VAD-free probability in the responder group (i.e., 89.3 ± 3.4%), this therapeutic option aimed to delay the need for HTx or MCS deserves much more attention. However, being costly, time-consuming, and requiring a multidisciplinary team with particular experience, IA is very difficult to implement. A possible alternative to IA could be the neutralization of AABs through the intravenous administration of small soluble molecules, such as peptides or aptamers, which specifically target and neutralize β1-AABs [26,39,40].

Non-ischemic DCM accounts for about 40% of all cardiomyopathies and is not only the most frequent initiator of HF in younger persons, but also the leading cause of HTx [10,13]. Given the large variety of possible causes which can ultimately contribute to the variable clinical presentation of non-ischemic DCM, its natural history remains incompletely understood [15,41,42]. In most instances, HF in patients with non-ischemic DCM results from an interplay between familial predisposition and direct myocardial damage caused by infectious, autoimmune, or toxic agents, as well as endocrine and metabolic abnormalities [9,10]. Severely impaired ventricular pump function related to structural and functional abnormalities of the contractile myocardium is the major cause of death in about 70% of the patients with chronic non-ischemic DCM [9]. DCM-specific spherical LV dilation as a consequence of myocardial remodeling and fibrosis leads, together with impaired myocardial contractile function, to a reduction in stroke volume (SV) and cardiac output (CO), as well as to diastolic dysfunction (i.e., impaired myocardial relaxation and reduced ventricular compliance) with impaired LV filling and high end-diastolic pressure [9,43]. The precise cause of LV myocardial fibrosis (MF), which has a continually increasing detrimental impact on LV function with the progression of non-ischemic DCM, is still disputed. There are, however, indications that the increasing wall stress causing direct mechanical injury and/or microvascular ischemia plus the activation of immune and neurohormonal axes leading to the production of profibrotic mediators (e.g., angiotensin II and aldosterone) can play a key role [44]. 

A major contribution to the progression of HF of different etiologies is attributed to the over-expression of biologically active molecules which can exert deleterious effects on the cardiovascular system [45]. The initial activation of the adrenergic nervous system (ANS) and renin–angiotensin system (RAS), which are responsible for maintaining systemic arterial blood pressure and CO (by enhancing myocardial contractility, inducing peripheral arterial vasoconstriction, and increasing the retention of salt and water) as well as the activation of inflammatory mediators responsible for myocardial repair and remodeling, can evolve from a compensatory response into a harmful pathogenic mechanism [45,46]. It is also known that some of the “classical” neurohormones (particularly norepinephrine and angiotensin II) are also synthesized within the myocardium, where they act in an authocrine and paracrine manner [45]. In particular, drugs that interfere with the excessive activation of the RAS and ANS can mitigate the symptoms of HF with reduced LVEF by stabilizing or even reversing the ventricular remodeling [43,44]. However, the sustained activation of neuro-hormonal and cytokine systems can lead to progressive maladaptive remodeling which will ultimately become sufficient to cause aggravation of the HF regardless of the neuro-humoral status of the patient [45]. This could explain the accelerated loss of the efficiency of angiotensin-converting enzyme inhibitors, angiotensin receptor blockers and β_1_-AR blockers, and other medications aimed to improve the neurohumoral status of patients with end-stage chronic HF.

**Figure 3:**
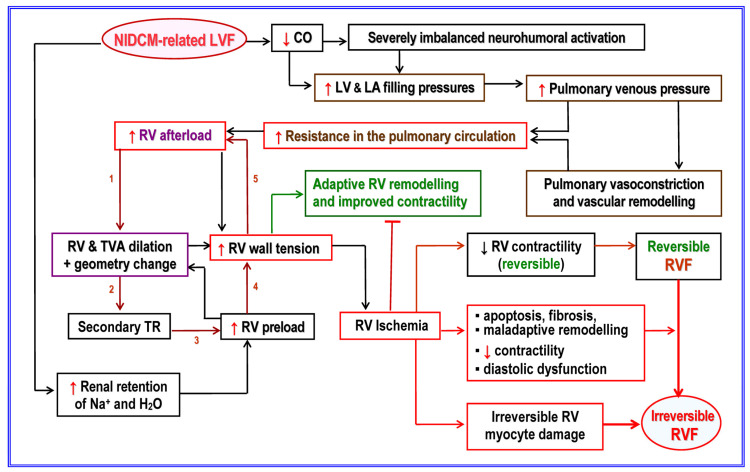
Pathophysiology of secondary right ventricular failure in patients with non-ischemic cardiomyopathy-induced left ventricular failure. NIDCM, non-ischemic dilated cardiomyopathy; LVF, left ventricular failure; CO, cardiac output; LA, left atrium; TVA, tricuspid valve annulus; RVF, right ventricular failure; TR, tricuspid regurgitation; ↑ and ↓, increase and decrease (reduction), respectively; ¯|¯ inhibit; the dark red arrows 1,2,3,4, and 5 describe the major vicious circle of RV overloading.

Of particular importance is the fact that end-stage HF involves both ventricles even if its initial cause was a left-sided heart disease [43]. Right HF is commonly related to LV dysfunction, and the high incidence of hemodynamic overloading-induced RV dilation and dysfunction in DCM with initial involvement of the LV is explained by the distinct load-dependency of RV size, geometry, and function [43,47]. Figure 3 provides an overview of the major pathophysiological mechanisms involved in the development of RV failure (RVF) secondary to NICM-triggered primary LV failure (LVF). 

### 2.2. Prediction of DCM-Related End-Stage Heart Failure

The course of HF and, in particular, that of the etiopathogenically heterogeneous non-ischemic DCM-related HF group is difficult to predict. For the prediction of death, the strongest and most consistently reported predictors were the total pulmonary vascular resistance (TPR), cardiac index (CI), renal function, brain natriuretic peptide (BNP) or N-terminal pro–B-type natriuretic peptide (NT-proBNP) levels, history of HF, patient age, and blood pressure [48,49]. Far more challenging appeared to be the prediction of DCM-related HF transition into its end stage, which can, however, play an even more crucial role in the optimal management of patients with symptomatic non-ischemic DCM. The difficult prediction of end-stage HF is quite understandable given the large variety of possible etiologies, pathophysiological particularities, and clinical presentations of the different DCM phenogroups which are summarized under the term non-ischemic DCM. Nevertheless, the reliable prediction of the imminent transition of a symptomatic HF to a refractory end-stage HF is of particular importance for potential HTx candidates for whom, in case of further severe clinical deterioration, HTx would be more advantageous than any durable MCS therapy. Given that the waiting time on HTx lists continues to increase, a timely prediction of the HF course could improve the patient prognosis by helping to avoid the need for implantation of a VAD support as BTT therapy. 

The difficulties in making accurate predictions about the short-term course of severe HF in potential HTx candidates rely primarily on the scarcity of studies relating to short-term prognostic markers in advanced non-ischemic DCM. In a short to medium-term (12 ± 9 months) follow-up study on 85 potential HTx candidates with DCM-related severe HF (LVEF <25%) without any significant differences neither in clinical |data, hemodynamic parameters, and standard echocardiographic (ECHO) parameters of LV size and function, nor in the survival rate between those without (83%) and those with coronary stenoses (17%), at the time of enrollment, only the baseline TPR and the CI were significantly different between the survivors and non survivors [49]. In that study, a TPR ≥14 Wood units and a CI <1.65 were the only independent predictors of prognosis on univariate and multivariate analysis, whereas neither the LV end-diastolic diameter (LVEDD) nor the LVEF revealed any predictive value [49]. In a short-term (six months) study which included 38 consecutive patients with idiopathic DCM referred for HTx, where 20 of them showed a life-threatening progression of HF (six patients died from HF, the other fourteen underwent VAD implantation), the standard ECHO-derived parameters of LV size (end-diastolic and end-systolic diameter), LV volume, and LV systolic function (i.e., LVEF) measured at the baseline were not significantly different between these patients and the other 18 patients who remained stable during the follow-up time [50]. The lack of significant differences at the baseline between the two patient groups was rather unexpected given the fact that the mean baseline NT-proBNP values were significantly higher in the unstable group (6058 pg/mL vs. 1950 pg/mL) [50]. Additionally, in another short-term (one year) study which included forty-five patients with DCM, where twenty-eight of them showed a life-threatening progression of HF leading to four cardiac deaths and twenty-four LVAD implantations, the LV size and LVEF were not significantly different between these patients and the other seventeen event-free patients [51]. These observations are of particular practical relevance because they question the reliability of conventional ECHO-derived LVEF and LVEDD to be used as parameters for decision making in the management of HTx candidates with non-ischemic DCM. From a pathophysiological point of view, the lack of predictive value revealed by LVEF and LVEDD is quite understandable because in the end stage of DCM-related HF they usually present mitral valve regurgitation (MR) related to the dilation of the mitral valve ring, which reduces the validity of LVEF for quantification of the LV systolic function, whereas the progressive reduction in LV compliance limits further LV dilation, corresponding to a progressive increase in LV filling pressures [52,53,54]. In a recent study, the new occurrence or worsening of MR in patients with DCM appeared independently predictive for their outcome, and the prognostic value of MR was incomparably higher than that of LVEF or LVEDD [55]. The authors also found that with aggravation of DCM-related LV dysfunction, the LVEF loses all of its predictive value [55]. This is absolutely understandable because, in patients with MR, the difference between end-diastolic and end-systolic volume is not solely the forward stroke volume (SV_f_), but the sum of SV_f_ and mitral regurgitant volume; with the aggravation of MR, the regurgitant fraction increases to the detriment of SV_f_ [54,56,57]. Thus, even patients with similar LV end-diastolic volume (LVEDV) and LVEF can have different SV_f_ measured at the LV outflow tract, and this suggests that patients with similar LVEF values but different degrees of MR can have significantly different degrees of LV dysfunction [54,57]. Among the conventional ECHO-derived measurements, the trans-mitral flow peak velocities of the E (early filling) and A (late filling caused by atrial contraction) waves, as well as the E/A ratio, were found more useful than the systolic parameters for predictions about the short-term course of HF in potential HTx candidates [50]. In a study by Jasaityte et al. [50], unstable patients referred for HTx had significantly higher E-wave velocities and E/A ratios, shorter E-wave deceleration times (DcT), and lower A-wave velocities. E/A values > 1.5 revealed the highest sensitivity, specificity, and predictive value of all their investigated conventional ECHO-derived diastolic parameters for short-term (six months) irreversible decompensation [50].

Echocardiographic strain-imaging-derived parameters appear to be more useful for the short-term prediction of DCM-related end-stage HF than conventional ECHO-derived parameters [50,58,59]. Jasaityte et al. [50] found that myocardial strain imaging using speckle-tracking can be particularly useful for the prediction of the short-term course of HF in potential HTx candidates. The baseline values of the global longitudinal and circumferential end-systolic strain (GLS and GCS), the longitudinal and circumferential intraventricular dyssynchrony indexes, the diastolic A-wave strain rate (DSR_A_), and the ratio between the E and A wave strain rates (DSR_E/A_) were found to be significantly different between patients who remained stable and those who died or required the emergent implantation of a VAD during the following six months [50]. The highest predictive values of all the investigated strain imaging parameters for short-term irreversible decompensation were the diastolic parameters DSR_A_ and DSR_E/A_ [50]. More recently, using 3D speckle-tracking imaging in addition to conventional ECHO, Rady et al. [59] demonstrated the existence of a close relationship between the alteration of LV torsion and the severity of HF in patients with non-ischemic DCM. That study, where the primary endpoint was hospital admission for HF worsening within 12 months of enrollment, indicated that impairment of LV torsion, which results from the opposing rotations of the LV base and apex during systole, has important prognostic relevance. Thus, whereas only 7% of the patients with relatively preserved LV torsion (>0.59 degrees/cm) necessitated short-term hospitalization, among the forty-five patients with reduced LV torsion, twenty-one (47%) necessitated frequent hospitalizations during which two patients died and another four underwent VAD implantation [59]. Univariate (unadjusted) Cox regression analyses identified, in addition to LV torsion, other ECHO parameters such as the LV GLS and GCS, the E/E’ ratio (i.e., ratio of pulsed-wave Doppler-derived early diastolic transmitral flow and tissue Doppler-derived early diastolic velocity of the lateral mitral annulus), the LV end-diastolic volume index, and TAPSE (i.e., tricuspid annular plane systolic excursion) as significant predictors. Nevertheless, after adjustment for age, NYHA class, and NT-proBNP, only LV torsion (*p* < 0.001) and TAPSE (*p* = 0.01) remained significant ECHO-derived predictors for one-year aggravation of HF [59]. Although Rady et al. [59] and Jasaityte et al. [50] included HF patients with different LVEF (i.e., <50% and <30%, respectively) in their studies, the LVEF appeared not to be a significant predictor for aggravation of HF in either study. Regarding the predictive value of TAPSE for the short-term progression of relevant HF, the aggravation of secondary tricuspid regurgitation (TR), triggered and further aggravated by the mainly pressure overload-induced RV dilation, can induce overestimations of RV contractile function by facilitating the RV free wall longitudinal motion, which increases the measured TAPSE corresponding to the increased blood volume leaving the RV in systole [60,61]. In another short-term study which included 61 patients with non-ischemic DCM (LVEF <30%) referred for HTx, where 29 events occurred (five patients died and another thirty-two underwent LVAD implantation), neither LVEF nor TAPSE revealed any reliable predictive value for the one-year outcome [62]. 

Given that increasing amounts of MF result in progressive LV stiffness and reduced LV compliance, inducing mainly diastolic, but also systolic, dysfunction, evaluation of MF by cardiac magnetic resonance (CMR) imaging can also be useful for the prognostic assessment of DCM-related symptomatic HF. In studies on patients with NICM (of different origins) and LVEF ≤35%, the presence and extent of MF assessed by CMR was strongly associated with an adverse cardiac prognosis [44,63,64]. It was also found that progressive MF has a particularly high predictive value for life-threatening aggravation of non-ischemic DCM [62]. Thus, in comparison with stable MF, progressive MF appeared to be associated with a more than three-fold higher risk for mortality and HF-related complications, even in patients with LVEF improvement after medical therapy [64]. As in ECHO studies, in CMR studies, the LVEF revealed no significant predictive value for life-threatening aggravation of HF in non-ischemic DCM [44,64]. 

The alteration of LV diastolic function and intraventricular synchronicity, the increase in total pulmonary vascular resistance, and the reduction in the CO and progressive alteration of right-sided heart geometry and function with the new appearance or rapid aggravation of preexistent TR were found to be important short-term prognostic markers [50,51,52]. Given that with the aggravation of HF, which was primarily initiated by left-sided heart dysfunction, the prognostic relevance of the secondary hemodynamic overload-induced morphological and functional right-sided heart alterations increases progressively; it is not surprising that for the short-term prediction of advanced DCM-related HF worsening, certain ECHO-derived right-sided heart parameters were often found to be even more reliable than many left-sided heart parameters [50,51,62]. In an ECHO study by Kawata et al. [62], the RV end-diastolic and end-systolic area indexes, as well as the fractional area change (FAC_RV_), appeared highly significantly associated with outcome in DCM patients referred for HTx. However it should be noted that the prevalence of TR ≥ moderate in both the event and non-event groups was low in that study (16% and 10%, respectively [62]) and there were no significant differences in the prevalence between the two patient groups (*p* = 0.43 [62]). Given that, with the worsening of the HF, both the prevalence and severity of TR will usually increase, the FAC_RV_ can become less reliable for the short-term prediction of life-threatening cardiac decompensation [65]. In their study, Xu et al. [51] identified the RV systolic/diastolic duration ratio (S/D_RV_) as the more useful conventional ECHO-derived parameter for the prediction of HF worsening in patients with non-ischemic DCM and LVEF <35% when compared with the LVEDV index, LVEF, eDcT, transmitral E/A ratio, left atrial volume index (LAV_i_), FAC_RV_, TR, and TAPSE. At a cutoff value of 1.2, the easily obtainable S/D_RV_, with the use of continuous Doppler imaging in the presence of ≥mild TR, revealed a sensitivity, specificity, and area under the curve of 79%, 65%, and 0.745, respectively [51]. As in other studies, the LV end-diastolic size and LVEF were not different between patients with and without cardiac events [51]. By adding an S/D_RV_ ratio >1.2 to the association between the conventional parameters BNP and LAV_i_, it was possible to obtain a significant incremental benefit for prediction of one-year events in patients with advanced non-ischemic DCM [51]. 

## 3. Patient Selection for Long-Term VAD Support

The decision for or against the implantation of a long-term MCS is based, in principle, on the presence of indications and the absence of contraindications [66]. It is accepted that patients considered for implantation of a long-term VAD support should meet the functional, hemodynamic, and clinical criteria for HTx candidate selection. The interplay between long-term VAD support and HTx depends largely on donor availability, allocation rules, and the potential changes in the risk profiles of the HTx candidates with or without VAD support while waiting for transplant. 

The major indications for the use of long-term VADs are: (1) as a BTT for patients listed for HTx who are “too sick” to wait for a donor heart, being at high risk of waitlist mortality and/or of impending secondary organ dysfunction; (2) as a bridge to candidacy for those who are not yet HTx candidates because of certain treatable or possibly only transient contraindications, but who might become eligible for HTx later; or (3) as a permanent lifelong support (so-called DT) for patients considered ineligible for HTx. [66,67,68,69,70]. Additionally long-term VADs can also be useful as a bridge to decision in patients at subacute high risk for HTx in whom prospective decision-making needs to be postponed for a longer period of time [66,70]. Finally, through restoration of optimal hemodynamics and reduction in ventricular wall tension, long-term VADs create favorable conditions for myocardial recovery, sometimes even enabling the transition of a VAD initially designed as a BTT or DT into a bridge to recovery (BTR) with VAD explantation as the end result [31,32,71,72,73]. However, given that long-term stable ventricular reverse remodeling and functional improvement after VAD implantation occurs rather rarely in patients with advanced chronic cardiomyopathy, VADs are not a risk-free therapy option, and cardiac recovery in these patients is currently not predictable before VAD implantation, implantation of a long-term VAD primarily designed as a BTR is not recommended for patients with NICM-related severe chronic HF [32,73]. Whereas in 2009, 80% of patients received their LVAD as a BTT, the leading indication for LVAD therapy is currently DT in patients who are ineligible for an HTx [66,70,74]. 

LVADs are basically indicated in inotrope-dependent HF patients with pure or predominant LV dysfunction. Although NICM-related end-stage HF involves both ventricles, particularly in patients with DCM, RV dysfunction is most often a secondary consequence of the LV failure-induced increase in the TPR [75,76]. Given that the mechanical support of the LV reduces the TPR, LVAD implantation is more often sufficient for a long-term mechanical support of the entire failing heart [43,75].

Optimal timing of VAD implantation is crucial but also particularly challenging, necessitating a multidisciplinary approach in experienced high-volume centers, preferably with a transplant background [70]. To reach this goal, a not yet absolutely necessary exposure to the risks of surgery and VAD-related complications (e.g., stroke, bleeding, infections and pump thrombosis) must be carefully weighed for each patient against the probability of dying or developing irreversible RV and/or end-organ dysfunction while delaying the device implantation [69,70]. There are several specific considerations of particular importance relating to risk–benefit assessment in candidates for a long-term VAD support, which are also valid for patients with NICM-induced chronic HF. In addition to the patient age and significant co-morbidities that may compromise outcomes, the risk–benefit evaluation must consider the presence of cardiac abnormalities such as patent foramen ovale; valvular abnormalities and thrombi; cardiac arrhythmias and aortic diseases (e.g., coarctation, atheroma, and aneurysma); alterations in RV size, geometry, and function; increases in the pre-capillary pulmonary vascular resistance; the existence of bleeding disorders; the presence of renal dysfunction; and the presence of acute or chronic infections [67,69,70]. Advanced age is not a contraindication to LVAD therapy per se, but the higher incidence of significant comorbidities may impair the outcome [67]. Decision-making for or against elective implantation of a permanent VAD is particularly challenging in patients over the age of 66 because the outcomes are often particularly poor in those with inotropic dependency or cardiogenic shock [77].

The introduction of the INTERMACS classification contributed substantially to the improvement of patient selection for long term LVAD support. Several studies indicate that, whereas implantation of a long-term LVAD should be considered in all INTERMACS 3 cases (stable on inotrope infusion), this therapeutic option can be considered only for carefully selected INTERMACS 1–2 (i.e., severe unstable inotrope-dependent) patients [67]. LVAD implantation could also be considered in severely symptomatic and motivated INTERMACS 4–7 (non-inotrope-dependent patients with different levels and types of advanced HF symptoms) patients who will accept a risk of adverse events in exchange for longer survival with a better functional status [65].

Untreated significant aortic valve regurgitation (AR) is a contraindication to LVAD implantation because it hampers both optimal organ perfusion and LV unloading by the LVAD. Elimination of significant AR at the time of LVAD implantation either by aortic valve (AV) replacement using a bioprosthesis or by closing the AV should be considered [67,78]. In stable patients, simultaneous AV replacement and LVAD implantation were not found to be associated with an impaired outcome [78]. However, in patients with cardiogenic shock, an additional AV replacement may impair outcome [78].

## 4. Biventricular Long-Term Mechanical Support

Despite the usual presence of some degree of RVF, and although the prevalence of RVF which complicated LVAD placement was found to range between 10% and 30%, over 90% of patients requiring long-term MCS can be successfully supported with an LVAD alone [69,79,80,81,82]. This is particularly true for patients with secondary RVF (hemodynamic failure due to increased RV afterload) triggered by an NICM-related chronic LVF with high postcapillary ± high precapillary pulmonary vascular resistance (PVR) [80]. 

Proper preoperative decision-making between the possibility of implanting a long-term LVAD only or the necessity of using a long-term biventricular assist device (BVAD) in a given patient is crucial because both unnecessary implantation of a BVAD (associated with more complications and lower quality of life) and irreversible RVF after LVAD implantation will significantly impair patient outcomes [7]. However, preoperative prediction of RV function after LVAD implantation is challenging and necessitates integrative evaluations of many different hemodynamic, ECHO, clinical, and laboratory parameters (including markers of neurohumoral activation and inflammation) [7,83,84]. Thus, whereas RVF due to irreversible RV alterations is preoperatively often predictable, RVF which becomes manifest only intra- or (early) postoperatively appears less predictable, while RVF which develops later after LVAD implantation proves to be most difficult to predict [7,84]. 

Although the support of a failing LV by an LVAD can normalize the left-side heart filling pressures and reduce the pulmonary venous pressure, thereby also reducing the postcapillary PVR, the severity and reversibility of both the LVF-related secondary increase in the precapillary PVR (generated by vasoconstriction and vascular remodelling) and the relevant RV remodeling and dysfunction, induced by the high afterload in the course of time, will remain decisive factors for the outcome of LVAD recipients [80]. In essence this means that before the decision-making in favor of either LVAD or BVAD implantation, there are two major questions that need to be answered. First, how much is the contribution of the pulmonary vascular disease triggered and aggravated by the upstream transmission of the high LA pressure? Second, how relevant is the impairment of RV adaptability to an increased pressure load induced by maladaptive RV remodeling ± irreversible functional alterations? Regarding the first question, the diastolic pulmonary gradient (DPG), defined as the difference between the invasive diastolic pulmonary artery pressure (dPAP) and mean pulmonary capillary wedge pressure (PCWP), enables the distinction between passive upstream transmission of LA pressure and increased PVR due to pulmonary vasoconstriction and/or pulmonary vascular structural changes [81,84]. Used as a surrogate marker of pulmonary vascular remodelling, DPG has proved helpful for predicting RVF in patients undergoing LVAD implantation, given that elevated DPG (≥7) in patients with end-stage HF requiring LVAD implantation appeared to be associated with increased risk of RVF [81]. 

In LVAD recipients with post-implant RVF, several echocardiographic parameters related to the right side of the heart were found to be significantly altered before LVAD implantation [85,86,87,88]. However, only a few of them, particularly the RA longitudinal strain, the RV free wall longitudinal strain, and the global peak systolic longitudinal strain rate, proved to be independent predictors of severe RVF requiring RV support [85,86,87].

The high load-dependency of RV geometry, size, and function requires the evaluation of the RV in relation to its current loading conditions using RV morphological and/or functional parameters in combination with pulmonary hemodynamic overload-reflecting parameters [84,89,90,91,92,93,94,95,96]. Among the right heart catheter (RHC)-derived combined parameters, the RV stroke work index (SWI_RV_), calculated as SWI_RV_ = (mean PAP − mean RA pressure) • SV_index_, and the pulmonary arterial pulsatility index (PAPi), calculated as PAPi = (PAPs − PAPd)/RAP, appeared particularly useful [91,92]. 

Regarding the ECHO-derived combined parameters, the pulmonary vascular capacitance (PVCAP), defined as SV/pulse pressure, the afterload corrected RV global peak systolic longitudinal strain rate (cPSSrL), and the RV load adaptation index (LAI_RV_) appeared particularly useful [84,96,97]. Their components are easy measurable, but the use of PVCAP is limited to patients with pulmonary regurgitation [95]. 

The cPSSrL, calculated as cPSSrL = PSSrL • ∆P_RV-RA_, where ∆P_RV-RA_ is the pressure gradient between the RV and RA (calculated from the mean velocity of the TR jet), is based on the relationship between RV longitudinal shortening velocity (i.e., a parameter of RV contractile function, which is more sensitive than the degree of shortening) and RV afterload [97]. Because the velocity of RV myocardial shorting is highly afterload-dependent, with the progressive LVF-induced increase in the PVR, the PSSrL decreases, whereas the ∆P_RV-RA_ increases as long as the RV can increase its systolic pressure to counteract the high resistance in the pulmonary circulation. Thus, the cPSSrL can remain relatively stable as long as the contractile reserves of the RV are not exhausted. However, once the afterload increase exceeds the ability of the RV to further increase its systolic pressure in order to prevent an SV reduction, in addition to the PSSrL reduction there will be also a reduction in the ∆P_RV-RA_ due to the increase in the RA pressure, which will result in a reduction in the cPSSrL. Once the adaptive capacity of the RV is exceeded, with a resulting reduction in the RV systolic pressure, the prognostic relevance of cPSSrL reduction substantially increases [97]. When compared with the RV free wall longitudinal strain, the cPSSrL was found capable of predicting RVF in patients undergoing LVAD implantation with substantially higher specificity and sensitivity [97,98]. The LAI_RV_ is another ECHO-derived, easily obtainable composite variable based, in principle, on the relationship between the ∆P_RV-RA_ and the RV end-diastolic size and geometry, expressed by the ratio between its end-diastolic volume (EDV) and the long-axis length (L_ED_). Using the TR velocity-time integral (VTI_TR_) instead of the ∆P_RV-RA_, which is unrestricted possibility and has the advantage of including the duration of systolic loading, and the RV end-diastolic area (A_ED_) instead of the EDV, the LAI_RV_, defined as:

                      VTI_TR_              VTI_TR_ (cm) • A_ED_ (cm)

LAI_RV_ = --------------  =  -----------------------------

     EDD/A_ED_            EDD (cm)

becomes a dimensionless and easily obtainable index reflecting the adaptation to the load.

Both the cPSSrL and the LAI_RV_ were found similarly useful for the prediction of RVF and lung transplant-free survival with severe pulmonary arterial hypertension (PAH) [99]. This, plus the fact that these two combined parameters were also found useful in improving the timing of listing to lung transplantation in patients with end-stage PAH [99], underpins the importance of cPSSrL and LAI_RV_ for the short- and medium-term prediction of irreversible RV remodeling and dysfunction. It is important to note that the predictive value of the LAI_RV_ appeared to be higher in LVAD candidates with non-ischemic cardiomyopathy than in those with ischemic cardiomyopathy. Thus, in a more recent study [100] which predominantly included patients with ischemic cardiomyopathy, although the predictive incremental prognostic value of the LAI_RV_ was significantly higher than that of the INTERMACS profiles and both the Michigan and European EUROMACS scores, and although the LAI_RV_ provided additional prognostic value to those validated risk profiles and scores, its predictive value was lower than in the Berlin study [97], which included predominantly LVAD candidates with NICM. Another possible explanation for the lower predictive value of the LAI_RV_ in the Stanford [100] study could be the higher average value of the LAI_RV_ in their patients (30.2 ± 11.3) when compared with those of the patients included in the Berlin study (median: 20 [8,9,10,11,12,13,14,15,16,17,18,19,20,21,22,23,24,25,26,27,28,29,30]) [97], which indicates the presence of patients with more altered RV size, geometry, and contractile function in the latter study. 

**Figure 4:**
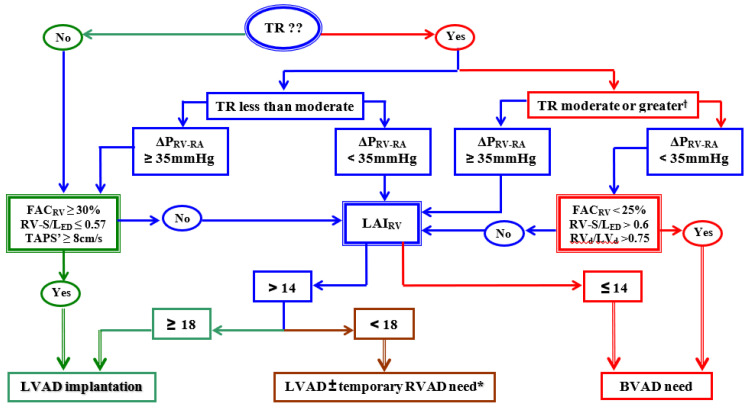
Transthoracic echocardiography-based algorithm for device selection in candidates for long-term ventricular assist device implantation. TR, tricuspid regurgitation; LV, left ventricle; ΔP_RV-RA_, pressure-gradient between right ventricle (RV) and right atrium (RA); FAC_RV_, RV fractional area change; S/L_ED_, end-diastolic short/long axis ratio; TAPS’, lateral tricuspid annulus peak systolic velocity; RV_d_/LV_d_, RV/LV diameter ration; LVAD, LV assist device, BVAD, biventricular assist device. * Because such algorithms are not yet validated by multi-center studies and especially because of the pathophysiological complexity of CHF-related RVF, they should be used only in connection with additional hemodynamic and clinical data. For decision making in favor of one or the other VAD-options, it is also useful to use both scoring-systems (which incorporate measures of different risk-factors for post-LVAD RVF) and composite variables (e.g., the LAI_RV_), which include RV geometry, function, and load; ^†^ >moderate TR might be considered for surgical repair at the time of implant.

As shown in Figure 4, the integrative evaluation of TTE-derived parameters of RV size, geometry, and function in relation to the current loading conditions of the right side of the heart can be particularly useful for device selection in candidates for long-term VAD [79,80,90,97,98,99]. Because, with only few exceptions, the numerous clearly confirmed risk factors for RVF after LVAD implantation are unable to reliably predict RV function during LVAD support, a pre- and intra-operative multi-parametric risk assessment for RVF evaluation appears indispensable [6,76]. Table 1 shows an overview of the combined variables, with the highest preoperative predictive values for RVF after LVAD implantation [79,97,98,101,102,103,104,105,106,107,108,109,110,111,112].

## 5. Cardiac Recovery during Long-Term Mechanical Support

By supporting and unloading the failing ventricle, VADs offer additional time and create favorable conditions for the reversal of structural and functional cardiac alterations. Abolishing the excessively increased ventricular wall tension and ameliorating the blood supply to the vital organs, VADs can deactivate major pathophysiological mechanisms involved in cardiac adverse remodelling, and even induce reverse remodelling accompanied by the clinically relevant reversal of cardiac structural and functional alterations. However, although during VAD support there is a high probability of reverse remodelling at cellular, molecular, and genomic levels; reversal of the HF phenotype is more often incomplete [84]. Thus, cardiac recovery enabling VAD explantation is much rarer than relevant myocardial improvement detectable at cellular and sub-cellular levels and appears to be especially related to the etiology of HF, patient age, the duration of HF, and the degree of myocardial fibrosis before VAD implantation [71,84,111,113,114,115]. 

After the first successful weaning from long-term LVAD, which was performed in 1994 in two patients who had recovered from acute HF [116], LVAD explantations were initially performed almost exclusively after myocardial recovery from acute forms of HF. This is not surprising, because acute HF reversal during mechanical LV support is not unusual, and the more frequent complete reversal of the myocardial alterations can facilitate the weaning decision-making [73]. Given that recovery from chronic HF is often incomplete (even in patients with long-term post-weaning freedom from HF recurrence) and the prediction of post-explant cardiac stability is particularly challenging, only a few centers have performed a relevant number of elective VAD explantations [31,71,117,118,119,120,121,122]. Thus, although the first elective LVAD explantations in patients with chronic HF were performed in Berlin in 1995 [31,32] and, at the end of 2011, the total number of patients with non-ischemic DCM who were weaned by the Berlin [117] and Harefield [122] groups from their long-term LVADs (initially designed as a BTT) increased to 53 and 37, respectively, until quite recently, chronic cardiac remodelling processes continued to be considered irreversible [73,123,124].

More than 15 years ago, it was demonstrated that weaning can provide at least the same chances of survival as HTx, even if HF recurrence may necessitate post-weaning HTx [71]. Meanwhile there is sufficient evidence that with the option of HTx for patients with post-weaning recurrence of HF, the survival rates of explanted LVAD recipients can be similar to those shown by patients transplanted after a BTT-LVAD support, and many of the weaned patients can achieve cardiac and physical functional capacities that are within the normal range of healthy controls [71,72,73,111,112,122,123,124].

### 5.1. Clinical Relevance of Chronic Heart Failure Reversal during VAD Support

Although full cardiac recovery is rather unusual in VAD recipients with pre-implant chronic HF, the systematic search for evidence of myocardial reverse remodelling and functional improvement is worthwhile because, for sufficiently recovered patients, weaning from VADs has proved to be feasible and able to provide survival benefits and better quality of life, even if recovery remains incomplete [71,72,73,111,122,123,124]. Studies revealed that with the option of HTx for those with recurrence of HF, the post-explant survival at one, three, five, seven, and ten years for patients with chronic NICM as the primary cause for the pre-implant HF can reach up to 90%, 78%, 74%, and 67%, respectively [117,122]. In a large study on patients with chronic NICM before VAD implantation, the five- and ten-year probability of post-weaning survival from HF recurrence or explantation-related complications (including the post-transplant survival time for patients with HF recurrence that needed HTx, but excluding non-cardiac-related causes of death during the follow-up period) reached about 88% and 83%, respectively [111,117]. 

For HF of different etiologies, and particularly for chronic NICM-related HF, a high range of recovery rates were reported [71,72,115,117,125,126,127,128,129,130]. This might be, in part, due to differences in the medical therapies which were used during VAD support, but are probably mainly related to the different selection criteria for VAD implantation and explantation used by different centers [31,71,117,120,125]. The incidence of myocardial improvement in long-term VAD recipients, which might enable weaning the patient off the mechanical support, should therefore be distinguished from very likely lower device explantation rates [119]. In adults with HF of different etiologies before VAD implantation, the reported overall cardiac recovery rate enabling VAD explantation was usually below 10% [125,126,127]. Of 1038 adults with end-stage HF of different etiologies who received a long-term VAD (BTT or DT) in the Berlin Heart Center between 1994 and 2011, a total of 96 (9.25%) were weaned from their VAD [117]. In the group of patients with chronic NICM, the recovery rate enabling explantation of the VAD reached 13.8% [117]. Also in Berlin, during the time when all weaned patients were supported by a pulsatile device (i.e., from 1994–2000) the explantation rate in the NICM group was much higher (up to 24%) [71]. This might suggest that patients with a pulsatile flow (PF) LVAD could have a much higher chance for myocardial recovery, possibly because PF devices may provide better unloading conditions for recovery [131,132]. However, considering the fact that, in Berlin, the preferential use of continuous flow (CF) VADs coincided with the implementation of more restricted criteria for VAD explantation, the assumed advantages of the PF systems become debatable. This is also supported by the fact that before the use of CF-VADs in Berlin, the rate of early post-weaning HF recurrence which necessitated HTx or a VAD reimplantation was considerably higher [71,117]. A recent prospective multicenter study which included LVAD recipients with NICM as the primary cause of their end-stage HF demonstrated that optimized mechanical unloading combined with a standardized reverse remodelling inducing specific pharmacological regimen and regular assessment of myocardial function can increase the incidence of cardiac recovery which enables LVAD explantation [129]. Thus, of the 40 studied patients, 19 (47.5%) underwent LVAD explantation and, during the first post-explant year, HF recurrence (necessitating HTx) occurred only in one of those patients. Post-explant patient survival at three years, without the necessity of HTx or implantation of another LVAD, reached 77% [129]. 

The overall VAD-promoted recovery rates reported for children ranged from 5–16.5% [133,134,135,136]. In a study from Berlin which included 147 children (age 6 ± 3 years, 52% with diagnosed DCM) supported with an EXCOR pediatric VAD (63% LVAD and 37% RVAD), 16.3% of them were weaned from the VAD after 78 ± 18 days of support [136]. The recovery rate in the LVAD and BVAD groups reached 20.6% and 9%, respectively [136]. Most important, however, was the fact that post-explant HF recurrence occurred in only one of the 24 weaned children, which, fortunately, could be successfully transplanted [136]. Thus, although cardiac recovery that enables VAD removal is quite rare even in children, for those who can be weaned from their VAD, the chances for long-term freedom from HF recurrence are generally better than in adults [134,135,136].

### 5.2. Optimization of Ventricular Support and Heart Failure Therapy

Providing the most optimal mechanical support for the LV is essential for the patient outcome. Pump speed should be chosen to provide sufficient CO while minimizing the load on the RV [137]. High pump speeds that cause leftward septal shift and subsequent increased TR or collapse of the left atrium or ventricle must be avoided [137]. Speed optimization in the early postoperative phase is determined by the interplay between antegrade flow through the LVAD, contractility of the heart, right and left ventricular filling pressures, and ventricular afterload. Thus, RV pressure overload with shifts of the interventricular septum to the left may require diuretic therapy, reduction in LVAD speed, and/or correction of systemic vasodilation to optimize LVAD function [137]. There are still controversies about the optimal speed settings for LVADs at discharge and during long-term patient management. Some practitioners prefer full LV support while others prefer partial support. The most appropriate long-term LVAD settings are likely not a simple recommendation for all patients and it should be considered a dynamic therapy which is dependent on patient-specific variables such as right heart function, left HF symptoms, and risk of gastrointestinal bleeding and AR [137,138]. LVAD speed adjustments to optimize unloading are likely to enhance the potential for recovery (Class IIa, level of evidence: C) [129,139,140]. 

Although in LVAD recipients, LV reverse remodelling might occur spontaneously, relevant remodelling occurs more often in patients with therapeutic interventions that either remove the initial stressor or mitigate some of the mechanisms that contribute to further deterioration of the failing heart, such as activation of the renin–angiotensin–aldosterone and adrenergic nervous systems [141]. Despite limited evidence, many clinicians use these agents in an attempt not only to treat hypertension, but also to maximize the chance of myocardial recovery. Hypertension leads to increased afterload for the LVAD, decreased LVAD flow, and less effective left ventricular unloading [142]. Angiotensin-converting enzyme inhibitors or angiotensin receptor blockers are recommended as the first-line drugs for hypertension. Beta-blockers can be used in combination with angiotensin-converting enzyme inhibitors or angiotensin receptor blockers, but caution is necessary in patients with marginal RV function [142]. Pharmacotherapy with neurohormonal blocking agents (angiotensin-converting enzyme inhibitors, angiotensin receptor blockers, angiotensin receptor blockers, neprilysin inhibitors, beta-blockers, mineralocorticoid receptor antagonists) is recommended for blood pressure management in durable LVAD patients (ISHLT Guidelines, Class I, level of evidence: B) [140]. 

Currently, it is recommended that all long-term VAD recipients with non-ischemic cardiomyopathy should be treated as potential bridge-to-recovery candidates [142]. The process of adverse remodelling can be stopped or even reversed with HF therapies, including angiotensin-converting enzyme (ACE) inhibitors, β-blockers, and aldosterone antagonists [143]. Although over the last years there has been important progress in the treatment of chronic HF, the standard anti-HF therapies have not been validated in the context of MCS [143]. Nevertheless, during ventricular unloading it appeared useful to treat all patients with beta-blockers, angiotensin-converting enzyme (ACE) inhibitors, aldosterone antagonists, and digitalis. Medication doses should be individually adapted to reduce the heart rate towards 60 beats/min and the blood pressure to the lowest optimally tolerated value that is also sufficient for maintaining optimal renal function. After detection of cardiac improvement which appears sufficient to consider the patient as a potential weaning candidate, in those with off-pump systemic arterial diastolic pressure values below 55 mmHg, it appears useful to reduce the doses of ACE inhibitors and diuretics in order to increase the diastolic pressure value up to 55–60 mmHg, which provides a more suitable afterload for the evaluation of LV systolic function during LVAD support interruption trials [144]. 

### 5.3. Detection of Potential Weaning Candidates 

Regular serial transthoracic ECHO (TTE) screenings are the first-line strategy for the detection of potential weaning candidates [71,123,140,144]. In hemodynamically stable patients, the screenings can be started after two to four weeks of optimal ventricular support [73,144]. During adequate and constant LVAD support, possible future weaning candidates are those with a sinus rhythm and normal heart rate, a regression of LV dilation toward normalization of LV size and geometry, a steady increase in both the frequency and duration of AV openings, an increase in LV wall motion amplitude (fractional shortening > 15%), no or maximum mild MR and/or AR, no relevant RV enlargement, and no or less than moderate TR [71,122,144,145,146].

### 5.4. Evaluation of Recovery 

The cornerstones of the diagnostic methods used to assess cardiac recovery in VAD recipients are ECHO and RHC. However, it must be considered that the reliability of both methods for weaning decision-making depends significantly on the degree of VAD support reduction achievable during the recovery evaluation trials, which necessitate repeated reductions in and short-term interruptions of the VAD support. 

TTE is the first-line strategy for the evaluation of recovery. [140]. In potential weaning candidates, before evaluating cardiac recovery during short-term interruptions of VAD support, it is important to carry out stepwise reductions of the ventricular support under TTE supervision to test if the necessary short-term complete interruptions of the mechanical support are already feasible [71,123,144,146,147] If such LV unloading reductions in support cause symptoms (such as dizziness or sweating) and/or cardiac dysrhythmias, or if the LV size increases above the normal range and/or the right-sided heart chambers reveal anatomical and/or functional instability (RV enlargement with decrease in its ejection, increasing TR, RA volume increase), the patient is not yet a weaning candidate and complete interruption of LVAD support is not advisable [117,140,148]. Nevertheless, the monitoring of these patients should be continued, and the support reduction test should be repeated after a few weeks [73]. Assessment of cardiac improvement and decision-making in favor of or against VAD explantation is mainly based on TTE- and RHC-derived measurements obtained during several short-time (≤15 min) interruptions of VAD support (pump stop or pump turn-down to zero unloading trials) under resting conditions and optimal anticoagulation [71,123,144,148,149,150]. PF-VADs enable recovery assessment during real off-pump trials (i.e., full deactivation of the VAD) [31,71,123,144,146,148]. Full deactivation of CF-LVADs can adversely affect the assessment of LV recovery. Thus, a complete stop allows a backflow of blood into the LV, which increases its size by volume overload and reduces the systemic arterial diastolic pressure (PAd), which in turn reduces the LV afterload [117]. These changes in LV preload and afterload can misleadingly affect both RHC and ECHO measurements. For CF-VAD recipients, lowering the rotor-speed (i.e., turn-down trial) to values resulting in ±0 pump flow in one cardiac cycle is therefore more useful than a total deactivation of these pumps [32,117,123,129,146,148,151,152,153]. The CF-LVAD speed necessary to suspend the ventricular support without any relevant retrograde blood flow into the LV depends on the pump design (e.g., 4000–6000 r/min for HeartMate II, 3000–4300 r/min for HeartMate 3, and 1800–2200 r/min for the HeartWare HVAD) [123,146,147,150]. Pulsed wave (PW) Doppler-derived calculations of forward and reverse velocity-time integrals at the inflow cannula can facilitate the achievement of zero net flow using pump speed adjustments [86,123,147,149,150]. RHC-derived hemodynamic measurements can also be affected by the retrograde blood flow initiated by complete stops of CF-LVADs [115,123]. This can be avoided by occlusion of the outflow cannula with an inflated balloon during the off-pump trials [86,117,123]. Such short-term balloon occlusions, which enable complete pump stops without any misleading retrograde flow into the LV, appear feasible and safe to perform [123]. 

The majority of patients who have been weaned from their VADs so far underwent recovery assessment exclusively at rest in order to avoid any detrimental impact of possible hemodynamic overloading on the often not yet finished myocardial recovery process [117,123,136,146,151]. Although recovery assessment at rest alone does not enable the evaluation of cardiac adaptation to physical demands, the outcomes of patients after VAD explantation were not worse than those reported by patients who additionally used dobutamine stress ECHO (DSE) and/or exercise testing for such an assessment [84,117,126,144,146,147,154,155,156]. Even though exercise stress ECHO, cardiopulmonary exercise testing during reduction or interruption of LVAD support, and off-pump DSE can provide useful details about the degree of myocardial recovery, the potential risk of myocardial overstraining associated with these tests (i.e., possible impairment of a still ongoing myocardial recovery processes) should not be disregarded [73,123,146]. The existence of such a risk is also supported by experimental studies which revealed that, after LVAD-promoted restoration of LV structure and function, recovered hearts are initially prone to hemodynamic stress, and that this vulnerability diminishes only over time [157]. 

**Figure 5:**
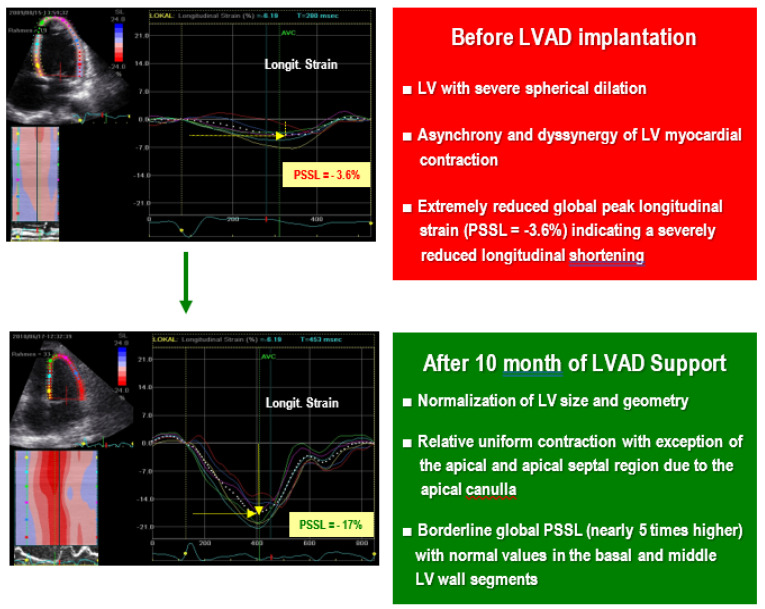
Longitudinal strain imaging for the assessment of mechanical unloading-promoted LV recovery after long-term LVAD support in a patient with advanced chronic non-ischemic cardiomyopathy.

ECHO examinations during LV support interruption trials (pump turn-down until zero-net flow or pump stop) should be performed step by step in the course of repeated short interruptions of the LVAD support for about 5–15 min [84,123,158]. The maximum permissible duration of the LV support discontinuations necessary to evaluate the extent of cardiac recovery is not precisely defined. In principle, multiple short (≤5 min) successive interruptions are less risky for the patient than one or two longer (15–20 min) cessations of the LV support [151,158]. Full pump stoppage or pump turn-down to ±0 flow should be considered very carefully in patients after strokes or transient ischemic attacks, as well as in those with hemolysis or problems with anticoagulation therapy, and are not indicated in patients suspected of pump thrombosis (even without LVAD dysfunction) [158]. Table 2 provides an overview of the most useful TTE-derived measurements for the assessment of cardiac improvement during off-pump (pump stop or pump turn-down) trials [32,59,86,98,117,123,136,138,139,140,141,142,143,144,145,146,147,148,150,152,159]. As shown in Figure 5, strain imaging can be particularly useful for the evaluation of LV functional improvement during short-term LV unloading interruption trials. 

Off-pump RHC becomes particularly important before any preliminary decision-making in potential weaning candidates with borderline TTE data and/or relevant cardiac improvement only after >6 months of unloading and/or a long history (>3 years) of HF [117,123,159]. A final off-pump trial of ≥15 min in the operation room, with repeated measurements of hemodynamic parameters under continuous ECHO monitoring, is mandatory before the start of explantation surgery [117,144]. 

Table 3 gives an overview of the most useful cardiocirculatory parameters for the assessment of cardiac improvement during off-pump trials in LVAD recipients [32,72,84,117,123,144,146,149,159,160].

ECHO and RHC are also the major tools for recovery assessment in BVAD recipients, but the whole procedure is more complex. To assess cardiac global recovery, it is necessary to interrupt the unloading of both ventricles. Given the high susceptibility of the RV to sudden increases in its loading conditions, the RV support should be stopped about 30 s before the interruption of LV unloading [80,117,144]. During interruption of the BVAD support, particular attention should be given to the RV size, geometry, and loading conditions, as well as to possible changes in TR. Increases in the RV end-diastolic diameter (RVEDD) and short-/long-axis ratio (L/S_RV_), as well as rises in right atrial pressure (RAP) beyond 10mmHg and increases in TR, indicate an inability of the RV to eject the necessary amount of blood into the pulmonary artery. The reason for that could be an inadequately recovered RV myocardial contractility or an increased resistance in the pulmonary vessels (inadequately recovered LV function), or also due to an inadequate myocardial recovery of both ventricles. If the PCWP is below 12 mmHg and the PAP remains unchanged or even falls, the explanation for the adverse right-sided heart response to the BVAD support discontinuation is an insufficient improvement of RV contractility. If, by contrast, right-sided heart dilation and RV dysfunction occur simultaneously with an increase in both PCWP (up to ≥13mmHg) and PAP, the major cause for that inadequate response to the BVAD discontinuation is a too-high RV afterload caused by an LVF-induced increase in left-sided heart filling pressures. If, in the absence of sufficient LV improvement, repeated discontinuations of only the RV support are not followed by pathologic changes in either the size, geometry, or filling pressures of the right-sided heart, nor in tricuspid valve competence and LV pump-flow, a switch from BVAD to LVAD support could be reasonable because RV improvement accompanied by a reduction in RV-supporting pump flow raises the risk of RV pump thrombosis [73,80].

### 5.5. Prediction of Cardiac Stability without VAD Support

The prediction of freedom from HF recurrence after LVAD removal in LVAD recipients with clear signs of myocardial improvement during mechanical support is even more challenging than the prediction of HF aggravation in potential HTx or LVAD candidates. Thus, whereas off-pump LVEF values from 35–45% in patients with chronic NICM are not predictive for irreversible cardiac decompensation, the vast majority of those who underwent LVAD explantation after attaining such EF values during the prolonged mechanical support experienced early recurrence of HF, necessitating HTx or further LVAD support [117,123]. As shown in Table 4 and Table 5, the pre-explant prediction of post-explant cardiac stability requires the integrative consideration of several cardiocirculatory parameters together with different factors that can affect or impact the value of those parameters. Thus, whereas off-pump LVEF ≥45% values in patients with normal off-pump RHC-derived hemodynamic parameters revealed only 74% positive predictive value (PV) for ≥5 year post-explant cardiac stability, and although the ECHO-derived LV size and geometry parameters (i.e., LVEDD and LV end-diastolic relative wall thickness [RWT_ED_], respectively) appeared not to be predictive for cardiac stability without LVAD support, the positive PV of LVEF ≥45% in combination with either LVEDD ≤ 55 mm or RWT_ED_ ≥0.38 can reach up to 86% and 87%, respectively (Table 4). Therefore, both LVEDD and RWT_ED_ measured by ECHO at the LV base in a parasternal long axis view [RWT_ED_ = (interventricular septum thickness + posterior wall thickness)/LVEDD] are indispensable for the reliable prediction of long-term post-weaning cardiac stability. Conversely, LVEF values ≥45% in patients without normalization of LV size (LVEDD >55 mm) and geometry (RWT_ED_ < 0.38) revealed a PV of 89% for HF recurrence during the first three years after LVAD explantation which was slightly higher than the 88% PV for early post-explant HF recurrence revealed by LVEF values of 35–45% in patients with complete reversal of LV size and geometry alterations [117,123]. In an earlier study, off-pump LVEDD values ≥56 mm revealed a 90% PV for recurrence of HF during the first three years after LVAD explantation [159]. 

Another important aspect is the high impact of HF duration until the need for a long-term VAD support. In larger studies on explanted patients with cardiac recovery of different degrees, HF duration of >3 years before LVAD implantation revealed a predictive value of 78% for HF recurrence during the first three years after LVAD explantation [32,117,159]. In a large study, among the explanted LVAD recipients who initially appeared to be sufficiently recovered from chronic NICM-induced HF, all those with a pre-explant off-pump LVEF between 35% and 45%, an LVEDD >55mm, and a pre-implant HF duration of ≥5 years needed an HTx or implantation of a second VAD before the end of the third post-explant year [159]. 

An often overlooked highly important aspect is the anatomic and functional cardiac stability after the VAD-promoted recovery has reached its maximum level. Because the extent of reverse remodeling and functional improvement, as well as the stability of those ameliorations, can vary greatly between the different patients, a careful evaluation of all parameter alteration during the course of each off-pump trial and also between the follow-up off-pump trials can be decisive for a successful weaning. Thus, whereas stable LVEF ≥45% after maximum improvement and during the final off-pump trial can reach a positive PV of 80% for ≥5 years post-explant freedom from HF recurrence, an unstable LVEF ≥45% (i.e., pre-explant alteration of >10% of the best LVEF value achieved before) can have a 90% PV for early (<3 years) post-explant HF recurrence necessitating HTx or implantation of another long-term VAD (Table 4 and Table 5) [117]. 

### 5.6. Decision-Making in Favor of Elective VAD *Explantation*

Because the most optimal duration of VAD support for the achievement of the maximum possible improvement in cardiac function varies widely among the VAD recipients, even if all of them had a NICM-induced HF before devise implantation, the VAD should be removed in recovered patients only after the follow-up off-pump tests show no further cardiac improvement [117,144]. Although weaning decision-making is primarily based on RHC and ECHO, neither of these two major diagnostic tools can reliably predict the post-weaning cardiac stability on its own. The precondition for a reliable prediction of post-explant cardiac stability using certain combinations of ECHO-derived anatomical and functional cardiac parameters is the normalization of the CI and both PCWP and RA pressures (i.e., >2.6 L/m², <13 mmHg, and <10 mmHg, respectively), proved by pre-explant RHC off-pump measurements [117,123,144]. PCWP increases above 13mmHg, reductions in CO by >15%, and a mean RA pressure >10 mmHg (or its increase by more than 50% during off-pump RHC measurements performed under resting conditions) are major risk factors for post-weaning HF recurrence [115,117,123,146]. 

Based on the experience gained to date, elective LVAD explantation can be considered in patients with sinus rhythm, normal and stable CI, PCWP, and mean RA pressure during the final off-pump trial, normal and stable off-pump LV size and geometry, stable LVEF ≥45% after maximum functional cardiac improvement between the pre-explant follow-up off-pump trials and during the final pre-explant off-pump trial, absence of or no relevant MR, AR, TR, or PR, as well as absence of RV dilation and/or relevant pulmonary hypertension during the final off-pump trial [117,123,146]. Conversely, unstable LVEF (reduction after maximum improvement as well as reduction during the final off-pump trial) with final off-pump values below 45% in patients with persistent spherical enlargement of the LV, MR >grade 1, RV dilation, and pre-implant HF duration ≥5 years can be considered contraindications for LVAD explantation, particularly in older patients or other patients who are less eligible for HTx [117,123,146]. Carefully selected clinically stable patients with stable sinus rhythm, off-pump LVEF values from 40–45%, normalization of off-pump LV size and geometry, no MR, normal and stable CI, PCWP, and mean RA pressure during the final off-pump trial, as well as less than five years of pre-implant HF could also be considered for elective LVAD removal [115,123,145,146]. Successful LVAD explantations in patients with off-pump LVEF ranging from 35–40% is an exception and is therefore not recommendable for elective LVAD explantation [71,115,123,145]. 

Given the multitude of factors involved in the maintenance of cardiac stability after VAD explantation, their different impact on cardiac anatomy and function, the high complexity of their possible interactions, as well as the usually incomplete functional recovery of the supported ventricle, weaning decision-making remains a difficult and challenging task. This also explains the lack of generally accepted guidelines for decision-making in favor of or against the elective removal of a durable mechanical ventricular support. The major steps for the evaluation of cardiac recovery and weaning decision-making in durable LVAD and BVAD recipients, based on ECHO and RHC off-pump examinations obtained in resting conditions, were recently described in a more detailed manner [123]. 

## 6. Conclusions and Future Tasks

In patients with NICM-related chronic HF, timely prediction of HF transition into its end stage, necessitating HTx or long-term VAD support as a BTT or DT, remains difficult and particularly challenging given the broad spectrum of possible etiologies, pathophysiological particularities, and clinical presentations of NICM. It is important to note that in patients with DCM, LVEF and LVEDD have not been proven to be useful for the prediction of HF’s transition to its end stage, whereas aggravation of MR and diastolic dysfunction, as well as increases in the TPR can provide useful prognostic information. Evaluation of MF by CMR imaging can also provide valuable information for the prognostic assessment of DCM-related HF. 

Decision-making between the possibilities of implanting only a long-term LVAD or the necessity of using a long-term BVAD for a patient is crucial because both unnecessary use of a BVAD and irreversible RVF after LVAD implantation will significantly impair patient outcomes. Pre-operative or, at the latest, intraoperative prediction of RV function after LVAD implantation is reliably possible, but necessitates integrative evaluations of many different hemodynamic, ECHO, clinical, and laboratory parameters. 

Based on the convincing weaning successes obtained in carefully selected VAD recipients with NICM as the underlying cause for the pre-implant end-stage HF, weaning from VADs can be considered as a validated and feasible clinical option, even if cardiac recovery remains incomplete. Although ventricular reverse remodeling and improvement of myocardial function during long-term VAD support enabling successful explantation of the device in patients with pre-implant HF caused by chronic NICM is relatively rare, the weaning results (survival, freedom from HF) can be equal or even greater than those after HTx, and the explanted patients are spared from the side effects of the immunosuppression therapy necessary after HTx. Therefore, ECHO-screenings for the detection of potential weaning candidates should be performed not only in LVAD recipients throughout the whole post-implant time, but also in BVAD recipients, although successful device explantations in the latter patients occur even more rarely. 

The most attractive potential indication for VADs could be their elective implantation as a therapeutic strategy for recovery in earlier stages of HF, when the reversibility of myocardial alterations is higher. This, however, necessitates a reliable prediction of VAD-promoted recovery before implantation, because VAD therapy is not a risk-free option. Unfortunately, although recovery the rates are higher in younger persons with shorter durations of HF, less ventricular dilation, and less fibrosis, neither the pre-implant LV size and EF nor the HF duration, patient age, or the absence of relevant fibrosis can predict the success of an elective VAD implantation as a BTR. 

There are two essential open questions for the future:

1. Can future research on molecular and cellular levels provide a platform for new adjunctive therapies (pharmacological and/or cell-based therapy, gene transfer, etc.) aimed to optimize recovery and increase the number of weaning candidates?
2. Can future research on cellular, molecular, and genomic levels provide data which might allow the detection of patients with the potential for recovery before VAD implantation?

## Figures and Tables

**Table 1:** Overview of the combined variables with the highest preoperative predictive values for right ventricular failure during LVAD support.

Combined or Integrative Variables (Units)	Components	AUC (95% CI*)	Cutoff Values	PV (%) for RVF	
				Positive	Negative
**TTE score (points [p])** [101]	FAC_RV_; LAV index; estimated RAP	0.73	≥5p	75.0	64.0
**SWI_RV_ (mm Hg • mL/m²)** [102,103]	SWI_RV_ = SV • (PAPm − CVP)/BSA	0.63	450	42.6	75.8
		(0.52–0.72)	300	72.2	54.9
**CVP/PCWP** [103,104]	Central venous pressure (CVP) Pulmonary capillary wedge pressure (PCWP)	0.68 (0.68–0.96) 0.82	0.63 -	64.5 -	53.3 -
**Michigan RVFRS** [p] [102]					
	Vasopressor requirement, Bilirubin ≥ 2 mg/dL, AST ≥80 IU/L, and Crea ≥ 2.3 mg/dL	0.73	≤3p	70.9	79.6
		(0.65–0.81)			
		-	≥5p	80.0	73.7
**Michigan RVFRS + RV/LV** [p] [102]	Michigan RVFRS components and RV/LV diameter ratio	0.74	-	-	-
**Michigan RVFRS + PSSL_RV_** [p] [98]	Michigan RVFRS components and RV peak systolic longitudinal strain	0.77	-	-	-
**RVF risk score for RVAD** **need** [p] [106]	Crea ≥ 1.9 mg/dL, CI ≤ 2.2L/min/m², SBP ≤ 96 mmHg, SWI_RV_ ≤ 250 mmHg • ml/m², severe RV dysfunction, and previous cardiac surgery	-	<30p	45.3	96.2
		-	≥65p	89.5	71.5
**RVF risk score** [p] [105]					
				
	PVR, inotrope dependency, obesity, destination therapy, ACE inhibitor and/or AT II receptor blocker therapy, and β-blocker therapy	0.74 ± 0.04	≥12p	83.3	69.3
		-	≤5p	56.0	88.9
**Quantitative preoperative risk score (CRITT)** [p] [108]	RV dysfunction, CVP, TR, tachycardia, and intubation	0.80	≥4p	80.0	-
		(0.72–0.88)			
		-	<2p	-	93.0
**Modified LV-echo-for-RV score** [p] [79]	LVEDD, LVEF, LAD/LVEDD, SWI_RV_, and serum bilirubin and albumin	0.79	≥7p	93.4	-
			≤3p	-	97.1
**RV load-corrected PSSrL (mmHg/s)** [89]	Peak systolic longitudinal strain rate (PSSrL) and pressure gradient between RV and RA (∆P_RV-RA_)	0.95	24	97	87
		(0.93–1.0)		(84–99)	(77–93)
**RV load adaptation index (LAI)** **(dimensionless)** [97]	TR velocity-time integral, RV end-diastolic area, and RV long axis length	0.97	14	83	97
		(0.97–0.99)		(73–88)	(95–99)
**Echo-derived RVGWE** [p] [99]	RV global strain, pulmonary pressure, and cardiac cycle timings	0.92	77%	sensitivity 100% specificity 82%	
**Pulmonary arterial pulsatility index (PAPi)** [109,110]	Pulmonary artery systolic pressure, pulmonary artery diastolic pressure, and right atrial pressure (RAP) PAPi = (PAPs − PAPd)/RAP	0.77 [101]	2	sensitivity 67% specificity 74%	
		0.94 [102]	1.85	sensitivity 94%, specificity 81%	
**Post-NTP PAPi combined with** **FAC and PAPs** [p] [110]	Post-sodium nitroprusside (NTP) pulmonary artery pulsatility index, fractional area change (FAC), and pulmonary artery systolic pressure (PAPs)	0.95 (0.89–1.0)	-	Predictive accuracy 90.7%	

LVAD, left ventricular assist device; AUC, area under the curve; CI*, confidence interval; PV, predictive value; RVF, right ventricular failure; TTE, transthoracic echocardiography; SWI_RV_, right ventricular stroke work index; FAC_RV_, RV fractional area change; LAV, left atrial volume; RAP, right atrial pressure; SV, stroke volume; PAPm, mean pulmonary arterial pressure; PAPs and PAPd, systolic and diastolic pulmonary arterial pressure, respectively; BSA, body surface area; RVFRS, RV failure risk score; AST, aspartate aminotransferase; Crea, creatinine; CI, cardiac index; SBP, systolic blood pressure; RVGWE, RV global work efficiency; PVR, pulmonary vascular resistance; ACE, angiotensin-converting enzyme; AT II, angiotensin II; TR, tricuspid valve regurgitation; LVEDD, LV end-diastolic diameter; LVEF, LV ejection fraction; LAD, left atrial diameter.

**Table 2:** Echocardiographic parameters with diagnostic and prognostic relevance for the selection of candidates for VAD support and/or for VAD recipients who become potential candidates for weaning from the mechanical support.

ECHO Modes	Relevant Measurements and Parameter Calculations
**M-mode and 2D** [32,68,86,117,123,136,144,146,147,148,150,152,159]	- Left ventricular end-diastolic and end-systolic diameter [LVEDD and LVESD, respectively]; - LV end-diastolic short/long axis ration [LV-S/L_ED_]; - LV end-diastolic relative wall thickness * [RWT_ED_ = (septum thickness + posterior wall thickness)/LVEDD]; - LV fractional shortening [FS] and LV ejection fraction [LVEF] measured by biplane Simpson’s method; - Right ventricular [RV] end-diastolic dimensions (measured on parasternal and apical views); - Tricuspid annular plane systolic excursion [TAPSE], measured by placing the M-mode tracer in the tricuspid annulus just against the RV free wall; - RV fractional area change [FAC] and ejection fraction [RVEF].
**Pulsed wave (PW) and Color Doppler** [32,68,86,117,123,136,144,146,147,148,150,152]	- Doppler indices of LV diastolic function (isovolumetric relaxation time, trans-mitral flow velocities); - LV stroke volume [SV] measured at the LV outflow tract; - Detection and severity grading of aortic, mitral, tricuspid, and pulmonary valve regurgitation.
**Continuous wave (CW) Doppler** [32,68,86,117,123,136,144,146,148,152]	- Pressure gradient between the RV and right atrium [∆P_RV-RA_] calculated from the mean velocity of the tricuspid valve regurgitation [TR] jet; - TR velocity-time integral [VTI_TR_] for calculation of the RV load adaptation index [LAI]; - Pulmonary artery systolic pressure estimation from the peak velocity of the TR jet;
**Pulsed Tissue Doppler (PW-TD)** [32,86,117,123,150]	- LV systolic wall motion peak velocity [Sm] at the basal posterior wall; - Mitral annulus early diastolic peak velocity [e’] for calculation of the E/e’ ratio; - Tricuspid lateral annulus peak systolic velocity [TAPS’];
**Speckle tracking** **2D and 3D Strain imaging** [59,86,98,117,123,159]	- LV global longitudinal and circumferential strain and strain rate; - Calculation of the afterload-corrected RV global peak systolic longitudinal strain rate [cPSSrL]; - Evaluation of LV synchrony and synergy of contraction. - LV torsion assessed by 3D strain imaging

M-mode, one-dimensional view of cardiac structures reflecting ultrasound waves along one ultrasound line; 2D, two-dimensional view; 3D, three-dimensional view. * measured at the basal LV in the long axis view.

**Table 3:** Overview of the most important cardiocirculatory parameters necessary for the assessment of cardiac improvement during off-pump trials in LVAD recipients [32,72,84,115,117,119,123,144,146,149,159,160].

Type of Investigation	Relevant Parameters and Threshold Values for Weaning Decision-Making
**Electrocardiography** [115,117]	- Sinus rhythm, heart rate (HR) <90/min - No more than 25% HR increase during off-pump trials
**Echocardiography** [117,123,144]	- LV end-diastolic diameter ≤55 mm (or ≤30 mm/m² BSA), stable after maximum improvement * - LV ejection fraction ≥45%, stable after maximum improvement * - Stable stroke volume (SV) during the final off-pump trial - Systolic wall motion peak velocity ^†^ (Sm) ≥8 cm/s, stable after maximum improvement - No or <grade II mitral and/or aortic valve regurgitation - No RV dilation (RVOT <35mm, end-diastolic short/long axis ratio <0.6) - No or ≤grade II tricuspid and/or pulmonary valve regurgitation
**Right Heart Catheterization** [72,115,117,123]	- Cardiac index (CI) >2.6 L/min/m² BSA - Pulmonary capillary wedge pressure (PCWP) <13 mmHg - Mean right atrial pressure (RAPm) < 10 mmHg - Diastolic pulmonary gradient (DPG) ^‡^ < 7 mmHg
**Arterial Pressure** [117,123]	- Mean systemic arterial pressure ≥ 65 mmHg during off-pump trials in CF-LVAD recipients

LV, left ventricle; BSA, body surface area; RV, right ventricle; RVOT, right ventricular outflow tract; CF-LVAD, continuous flow left ventricular assist device. * stable values during and between the pre-explant off-pump trials; ^†^ measured with the pulsed tissue Doppler at the basal posterior wall in the LV long axis view; ^‡^ DPG, diastolic pulmonary artery pressure—PCWP.

**Table 4:** Relevance of major individual risk factors and different constellations of risk factors for heart failure recurrence after LVAD explantation in patients with non-ischemic chronic cardiomyopathy which caused the need for a mechanical circulatory support [32,72,84,117,123,144,146,149,159,160].

Pre-Explant Measurements at Off-Pump Trials			PV for ≥5 Years Post-Weaning Cardiac Stability [30,108,129]
Examination	Parameters and Threshold Value	Measurement Details	
**ECHO**	**LVEF ≥45%**	- measured at the last off-pump trial - stable from maximum improvement to LVAD explantation	74% 86%
	**LVEF ≥45% plus LVEDD ≤55 mm**	- measured at the last off-pump trial - both LVEF and LVEDD stable from maximum improvement to LVAD explantation	86% 94%
	**LVEF ≥45% plus RWT_ED_ ≥0.38**	- measured at the last off-pump trial	87%
	**LVEF ≥45% plus Sm ≥8 cm/s**	- both LVEF and Sm stable from maximum improvement to LVAD explantation	87%
	**SV (PW Doppler-derived VTI in the LVOT)** **Absence or ≤ grade 1 AR and/or MR** **No RV dilation (RVOT <35 mm, S/L <0.6)** **Absence or ≤ grade 2 TR and/or PR**	- stable during the pre-explant off-pump trials - assessed at the last off-pump trial - assessed at the last off-pump trial - assessed at the last off-pump trial	All are required preconditions for successful weaning. Alone, none of these parameters can predict post-weaning cardiac stability.
**RHC**	**Cardiac index (CI) >2.6 L/m² BSA** **PCWP <13 mmHg** **RAP <10 mmHg**	- measured during the last off-pump trial - measured during the last off-pump trial - measured during the last off-pump trial	RHC alone cannot predict post-weaning cardiac stability, but the limit values are required preconditions for successful weaning.

ECHO, echocardiography; RHC, right heart catheterization; PV, predictive value; LVEF, left ventricular ejection fraction; LVEDD, left ventricular end-diastolic diameter; RWT_ED_, LV end-diastolic relative wall thickness; Sm, LV systolic wall motion peak velocity; SV, stroke volume; VTI, velocity–time integral; LVOT, LV outflow tract; AR, aortic regurgitation; MR, mitral regurgitation; RV, right ventricle; RVOT, RV outflow tract; TR, tricuspid regurgitation; CI, cardiac index; BSA, body surface area; PCWP, capillary wedge pressure; RAP, right arterial pressure.

**Table 5:** Prognostic relevance of the major risk factors for heart failure recurrence after LVAD explantation in patient with evidence of cardiac reverse remodeling and functional improvement during follow-up off-pump trials [32,72,84,117,123,144,146,149,159,160].

Risk Factors for Heart Failure * (HF) Recurrence after Left Ventricular Assist Device (LVAD) Explantation	PV for HF Recurrence during the First 3 Years after LVAD Explantation [30,108,129,143,145]
Pre-implant HF duration >3 years	78%
Left ventricular ejection fraction (LVEF) 35–45% at the final pre-explant off-pump trial	88%
LVEF 35–45% at the final pre-explant off-pump trial in patients with pre-implant HF duration of >5 years	100%
LVEF ≥45% but unstable (i.e., pre-explant alteration of >10% of the best LVEF value achieved until then)	90%
LV end-diastolic diameter (LVEDD) 56–66 mm	90%
No LVEDD normalization plus persistence of LV geometry alterations (RWT_ED_ <0.38) despite of optimal LVEF (≥45%)	89%
LVEF ≥45% but unstable geometry (RWT_ED_ reduction of >8%, or S/L_ED_ increase of >10% at the final off-pump trial)	87% and 85%, respectively
Unstable LVEF ≥45% with either reduced wall motion velocity (Sm < 8 cm/s) or unstable Sm (alteration of >10% during the final off-pump trial)	83% and 90%, respectively
SV reduction (i.e., VTI reduction in the LVOT during the final off-pump trial)	All these parameter abnormalities are validated risk factors for early recurrence of HF after LVAD explantation. Precise information on their predictive value for early recurrence of HF after LVAD removal is currently not available.
Relevant LV diastolic stiffness despite optimal LVEF ≥45%	
LVEF 45–50% with concomitant MR grade 1–2 which can induce misleading overestimation of LVEF	
Systemic AP_d_ ≤ 50mmHg (can misleadingly increase the LVEF)	
Asynchrony or dyssynergy of LV contraction at the final off-pump trial	
RV size and geometry alteration, and/or a reduced LAI_RV_ during the final off-pump trial	
TR new appearance or aggravation with or without jet velocity increase during the final off-pump trial.	

* heart failure induced by a chronic non-ischemic cardiomyopathy; PV, predictive value; RWT_ED_, LV relative wall thickness; S/L_ED_, LV end-diastolic short/long axis ratio; Sm, systolic peak wall motion velocity measured at the basal posterior wall; SV, stroke volume; VTI, velocity-time integral; LVOT, LV outflow tract; MR, mitral regurgitation; AP_d_, systemic arterial diastolic blood pressure; RV, right ventricle; LAI_RV_, right ventricular load adaptation index; TR, tricuspid regurgitation.

## Data Availability

The review article contains no unpublished research data.
